# Effects of Deep Neuromuscular Block during Robot-Assisted Transaxillary Thyroidectomy: A Randomized Controlled Trial

**DOI:** 10.3390/jcm12113633

**Published:** 2023-05-23

**Authors:** Myung Il Bae, Sang-Wook Kang, Jong Seok Lee, Na Young Kim, Bahn Lee, Gilseong Moon, Young Chul Yoo, Kee-Hyun Nam

**Affiliations:** 1 Department of Anesthesiology and Pain Medicine, Anesthesia and Pain Research Institute, Yonsei University College of Medicine, Seoul 03722, Republic of Korea; 2 Department of Surgery, Yonsei University College of Medicine, Seoul 03722, Republic of Korea

**Keywords:** robot-assisted transaxillary thyroidectomy, deep neuromuscular block, postoperative pain, postoperative paresthesia

## Abstract

The occurrence of significant pain and paresthesia after robot-assisted transaxillary thyroidectomy has been reported, and some patients experience chronic symptoms even three months after surgery. This study scrutinized the effects of deep neuromuscular block during robot-assisted transaxillary thyroidectomy on postoperative pain and sensory changes. In this single-blinded, prospective, randomized, controlled trial, 88 patients who underwent robot-assisted transaxillary thyroidectomy were enrolled and randomly allocated to either the moderate or deep neuromuscular block groups. Study endpoints included postoperative pain, paresthesia, and sensory change after surgery. The linear mixed models for numeric rating scale pain scores in the chest, neck, and axilla all showed significant intergroup differences over time (*p* = 0.003 in chest; *p* = 0.001 in neck; *p* = 0.002 in axilla). In the post hoc analysis with Bonferroni correction, the pain scores of the chest, neck, and axilla were significantly lower in the deep neuromuscular block group on postoperative day one compared to the moderate neuromuscular block group (adjusted *p* < 0.001 in chest, neck, and axilla). This study demonstrated that deep neuromuscular block could reduce postoperative pain after robot-assisted transaxillary thyroidectomy. However, it could not demonstrate that deep neuromuscular block reduces paresthesia or hypoesthesia after the surgery.

## 1. Introduction

Robot-assisted transaxillary thyroidectomy (RATT) has gained popularity in recent years and offers many advantages over conventional open surgery, including improved cosmetic satisfaction without any difference in cancer control or safety [1,2,3]. However, several studies have reported the occurrence of significant pain and paresthesia after RATT, and some patients experience chronic symptoms even three months after surgery [4,5,6,7]. Many studies have attempted to mitigate the postoperative pain and sensory changes, but a definitive solution has not yet been found.

**Figure 1:**
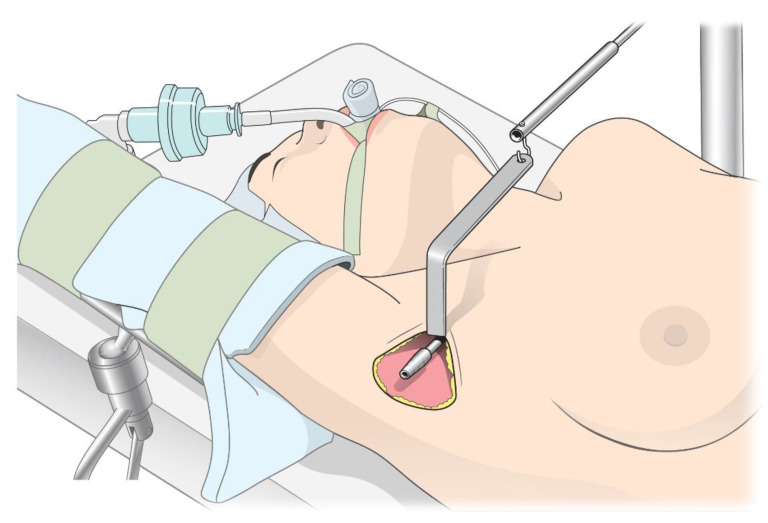
Description of chest skin flap elevation in robot-assisted transaxillary thyroidectomy.

The surgical procedure for RATT includes chest skin flap elevation by using an external retractor system to form a working space [6,8,9] (Figure 1). During this procedure, significant pressure is applied to the skin flap, and this pressure is presumably associated with postoperative pain and sensory changes [5,10]. Assuming that complete muscle relaxation could enhance visualization and reduce the retractor pressure required for skin flap elevation, deep neuromuscular block (NMB) may help reduce postoperative pain and sensory changes. In laparoscopic abdominal surgery, deep NMB has been demonstrated to mitigate postoperative pain and improve surgical conditions [11,12,13]. Nevertheless, its effect on RATT remains unknown. Therefore, we scrutinized the effect of deep NMB during RATT on postoperative pain and sensory changes. We hypothesized that deep NMB reduces postoperative pain and sensory changes after RATT.

## 2. Materials and Methods

### 2.1. Patient Enrollment and Randomization

This study was designed as a single-blinded, prospective, randomized, controlled trial. The Institutional Review Board and Hospital Research Ethics Committee of the Severance Hospital at Yonsei University College of Medicine approved this study on 27 November 2018 (#4-2018-0963). It was registered at ClinicalTrials.gov (NCT03871387) on 12 March 2019. We obtained written, informed consent from all participants. The study was conducted in accordance with the Declaration of Helsinki.

We included patients aged 20–70 years who underwent RATT and had an American Society of Anesthesiologists physical status of I–III. The following patients were excluded: (1) patients scheduled for radical neck node dissection; (2) those undergoing simultaneous surgeries for other organs; (3) those with other accompanying cancers; (4) those with a BMI > 30 kg/m^2^; (5) pregnant or lactating women; (6) those with a history of liver or renal failure; (7) those with a history of allergic reaction to rocuronium or sugammadex; (8) those who already had chronic pain or paresthesia; (9) those who could not understand the consent form, such as people with low levels of literacy or foreigners.

The enrolled patients were randomly allocated to the moderate NMB group or the deep NMB group. Group allocation was performed via a predetermined randomization sequence, which was created by a computer-generated random table without any block or stratification. The random sequence was kept enclosed in an envelope. A predetermined investigator was responsible for random sequence security and group assignment, and that investigator did not participate in the subsequent research process. The patients, surgeons, and investigators who evaluated the outcomes were blinded to the group allocation. However, assignment results were disclosed to the attending anesthesiologists to control the level of NMB according to groups.

### 2.2. Perioperative Management and Study Protocol

When the patient arrived in the operation room, non-invasive blood pressure, electrocardiography, pulse oximetry, and bispectral index (BIS, Covidien, Dublin, Ireland) monitoring were initiated. A TOF-Watch^®^ SX (Organon Ltd., Dublin, Ireland) was attached to the patient’s wrist. Glycopyrrolate 0.1 mg was administered intravenously before induction. Continuous remifentanil infusion was initiated at a rate of 0.05 to 0.2 μg/kg/min, and 1.5 to 2 mg/kg of propofol was administered intravenously. The TOF-Watch^®^ SX was calibrated when the patient became unconscious. After calibrating the device, 0.6 mg/kg of rocuronium was administered to the patient, and endotracheal intubation was performed. Anesthesia was maintained with desflurane and a continuous infusion of remifentanil. All surgeries were performed by three experienced surgeons (K.H. Nam, S.W. Kang, and C.R. Lee) who were blinded to the group allocation.

Rocuronium was continuously infused during surgery, and the infusion rate was adjusted differently for each group. For the moderate NMB group, the rate of rocuronium infusion was adjusted to maintain one to two responses in the train-of-four (TOF). For the deep NMB group, the infusion rate was adjusted to maintain zero responses in the TOF and one to two responses in the post-tetanic count [14]. The TOF-Watch^®^ SX on the patient’s wrist was used to monitor the TOF and post-tetanic count [15]. The attending anesthesiologists had been previously trained in controlling the level of NMB during surgery.

Before the end of the surgery, 1 g of propacetamol and 0.075 mg of palonosetron were administered. When the surgery was completed, sugammadex was administered intravenously (2 mg/kg for patients showing 2 or more TOF responses, and 4 mg/kg for those showing fewer than 2 TOF responses), and the investigator measured the time from sugammadex injection to a TOF ratio of 0.9. After the time was measured, desflurane and remifentanil were discontinued, and the endotracheal tube was removed when spontaneous respiration recovered. Patients who complained of pain at the post-anesthesia care unit (PACU) were administered 50 mg of tramadol, and those who complained of nausea were administered 0.3 mg of ramosetron. Pain control in the ward was conducted following the routine protocol of our hospital. On the day of surgery, 400 mg of ibuprofen was administered intravenously twice a day. From postoperative day (POD) 1 to POD 3, 200 mg of ibuprofen was routinely administered orally thrice daily. For patients requiring additional analgesia, 1 g of acetaminophen or 400 mg of ibuprofen was additionally administered intravenously.

### 2.3. Study Endpoints

The primary endpoint was the numeric rating scale (NRS) scores for pain on POD 1. The secondary endpoints included (1) NRS scores for postoperative pain until three months after surgery, (2) postoperative paresthesia, (3) postoperative sensory changes, (4) postoperative nausea and vomiting, (5) time from sugammadex injection to TOF ratio of 0.9, and (6) postoperative complications.

Postoperative pain was evaluated by using an NRS score of 0–10 (where 0 = no pain, and 10 = severe pain) with a single measurement. The pain scores were separately evaluated for the neck, chest, and axilla and were evaluated at PACU, POD 1, POD 3, and 3 months after surgery (POD 90). Paresthesia of the neck and chest was evaluated on a scale of 0–7 (where 0 = no paresthesia, and 7 = severe paresthesia) by using a questionnaire on POD 1, POD 3, and POD 90 [16]. Additionally, the degree of remnant sensation on the surgical site was evaluated by using the pinprick test on POD 1, POD 3, and POD 90. For the pinprick test, the investigator pricked the patients’ neck and chest on the surgical site with a 256 mN filament, and the patients were asked to rate the sensation in the pricked area as a percentage of the sensation on the opposite side of the chest (non-surgical site). Postoperative nausea and vomiting were assessed on a scale of 0–3 (where 0 = no nausea; 1 = mild nausea that subsided without medication; 2 = severe nausea requiring medication; and 3 = retching or vomiting) at PACU, POD 1, and POD 3 [17].

In the PACU, endpoints were evaluated after being admitted to the PACU and measuring vital signs. On POD 1 and POD 3, endpoints were measured in the morning just before administration of routine oral analgesics. On POD 90, endpoints were measured when the patient visited the outpatient clinic for follow-up. All endpoint measurements were performed by investigators who were blinded to the group allocation.

### 2.4. Sample Size and Statistical Analysis

A previous study reported a visual analogue scale pain score of 3.04 ± 1.28 on POD 1 after RATT [18]. Under the assumption that deep NMB could decrease the visual analogue scale score by 30%, the number of participants required to obtain a power of 90% was 44 in each group, considering a type 1 error (α) of 0.05 and a drop-out rate of 5%.

We used SAS software version 9.4 (SAS Inc., Cary, NC, USA) and IBM SPSS Statistics version 25.0 (IBM Corp., Armonk, NY, USA) for statistical analysis. We performed the independent t-test or Mann–Whitney U test in comparing the continuous variables, and we conducted chi-square test or Fisher’s exact test in comparing the categorical variables. The NRS pain score, paresthesia score, and degree of remnant sensation were analyzed by using linear mixed models, and post hoc analysis was conducted with Bonferroni correction.

## 3. Results

**Figure 2:**
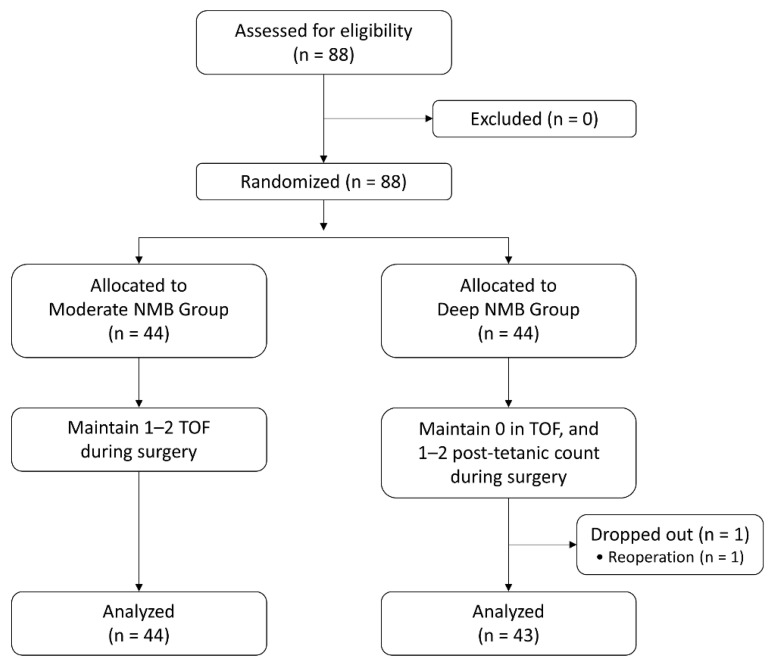
Flow diagram of the study. NMB: neuromuscular block; TOF: train-of-four.

In total, 88 patients were enrolled in the current study, and the patients were evenly allocated to the moderate NMB group (n = 44) or the deep NMB group (n = 44). Two cases of postoperative bleeding occurred in the deep NMB group; one of them underwent reoperation and was dropped from the study, and the other patient received only conservative management and was included in the analysis. Finally, 87 patients were included in the analysis (Figure 2). There were no missing values in the hospitalization data; however, data for POD 90 had missing values in 18 patients.

No significant intergroup differences were observed in demographic and clinical characteristics (Table 1). There were no significant intergroup differences in operation type, surgeon, tumor size, and pathology result (Table 2). Rocuronium and sugammadex were administered in significantly larger doses in the deep NMB group than in the moderate NMB group (rocuronium, 57 vs. 110 mg, *p* < 0.001; sugammadex, 121 vs. 244 mg, *p* < 0.001). The vital signs and peak airway pressure did not show significant intergroup differences, except for the heat rate when the robot was undocked. The time from sugammadex injection to TOF ratio of 0.9 was significantly longer in the deep NMB group than that in the moderate NMB group (99 vs. 147 s, *p* = 0.001). PACU time and length of hospital stay did not significantly differ between the two groups. There was no significant difference in the number of patients who received rescue analgesics between the two groups. No patients experienced respiratory failure or desaturation after surgery (Table 3).

The deep NMB group showed significantly lower NRS pain scores of the chest than that in the moderate NMB group immediately after surgery (in PACU) and on POD 1 and POD 3 (4.3 vs. 3.0 in PACU, *p* = 0.020; 3.6 vs. 1.6 on POD 1, *p* < 0.001; 2.0 vs. 1.0 on POD 3, *p* = 0.007). The deep NMB group also showed significantly lower NRS pain scores of the neck and axilla on POD 1 compared to that in the moderate NMB group (Supplementary Table S1). The number of patients complaining of severe chest pain with NRS > 6 in the PACU was significantly higher in the moderate NMB group than that in the deep NMB group (29.5% vs. 11.6%, *p* = 0.039). Additionally, the number of patients complaining of severe axilla pain on POD 1 was significantly higher in the moderate NMB group than that in the deep NMB group (13.6 % vs. 0%, *p* = 0.026) (Supplementary Table S2).

**Figure 3:**
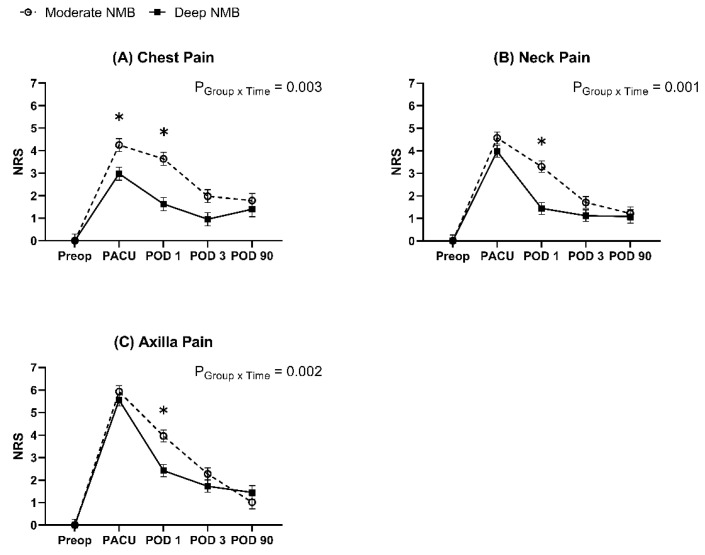
Changes in pain scores. The graph represents the changes in the estimated mean pain scores by linear mixed models, and error bars represent standard errors. Pain scores are expressed on a numeric rating scale of 0–10 (0 = no pain and 10 = severe pain). * Bonferroni-corrected *p* < 0.05 compared to the moderate NMB group. NRS: numeric rating scale; NMB: neuromuscular block; Preop: preoperative; PACU: post-anesthesia care unit; POD: postoperative day.

The linear mixed models for NRS pain scores in the chest, neck, and axilla all showed significant intergroup differences over time (*p* = 0.003 in the chest; *p* = 0.001 in the neck; *p* = 0.002 in the axilla). Post hoc analysis with Bonferroni correction revealed that the NRS pain scores of the chest, neck, and axilla on POD 1 were significantly lower in the deep NMB group compared to those in the moderate NMB group (3.6 vs. 1.6 in chest, adjusted *p* < 0.001; 3.3 vs. 1.4 in neck, adjusted *p* < 0.001; 4.0 vs. 2.4 in axilla, adjusted *p* < 0.001) (Figure 3).

**Figure 4:**
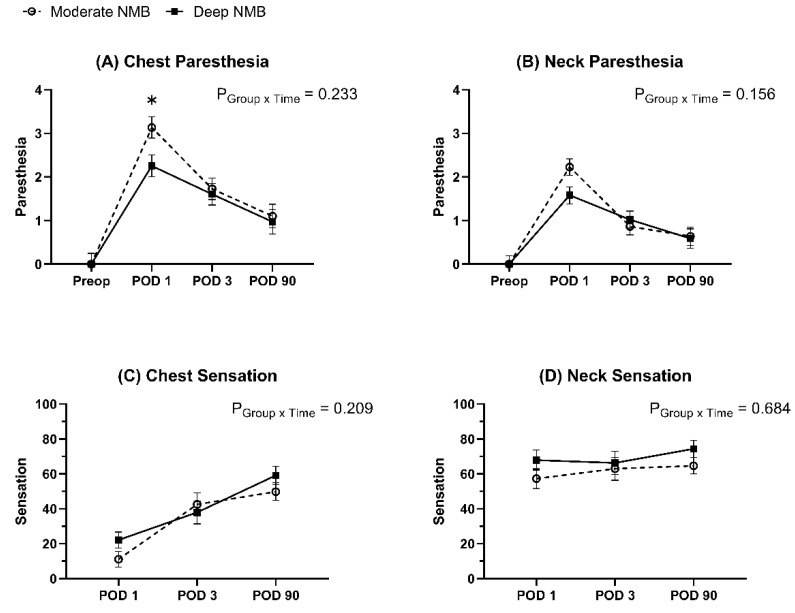
Changes in paresthesia scores and remnant sensation. The graph represented the changes in the estimated mean of paresthesia scores and the estimated mean of remnant sensation by linear mixed models. Error bars represented standard errors. Paresthesia scores were expressed on a scale of 0–7 (0 = no paresthesia and 7 = severe paresthesia), and the degree of remnant sensation was expressed as a percentage of 0–100%. (0 = no sensation, 100% = complete sensation). * Bonferroni-corrected *p* < 0.05 compared to the moderate NMB group. NMB: neuromuscular block; Preop: preoperative; PACU: post-anesthesia care unit; POD: postoperative day.

The linear mixed models for paresthesia scores and degree of remnant sensation did not show intergroup differences over time. Post hoc analysis revealed that the paresthesia score of the chest on POD 1 was significantly lower in the deep NMB group compared to that in the moderate NMB group. (3.1 vs. 2.3, adjusted *p* = 0.049) (Figure 4).

## 4. Discussion

This was the first investigation on the effects of deep NMB in RATT. This study demonstrated that deep NMB could reduce postoperative pain after RATT; however, it could not demonstrate that deep NMB reduces paresthesia or hypoesthesia after surgery.

The mechanisms underlying pain and sensory changes after RATT remain unknown. However, some researchers have argued that the retractor system may be involved [5,10]. RATT includes skin flap formation over the pectoralis major muscle, followed by flap elevation by using an external retractor system to form a working space without carbon dioxide insufflation [6,8,9] (Figure 1). However, vigorous flap elevation exerts significant pressure on the skin flap, and this may cause tissue damage or nerve injuries in the anterior chest [5,10]. It is well known that nerve injury causes neuropathic pain or paresthesia through nociceptor sensitization [19,20,21], and tissue damage also causes neuropathic pain or hyperalgesia by releasing various inflammatory mediators [19,22,23]. We speculated that lowering the retractor pressure in RATT could reduce tissue damage or nerve injury, mitigating postoperative pain and sensory change. One of the factors responsible for the high retractor pressure is insufficient muscle relaxation, and previous studies have reported that deep NMB significantly lowers the retractor pressure during spinal surgery [24]. Deep NMB presumably lowers the retractor pressure in RATT by enabling sufficient relaxation of the muscles in the neck and chest. As expected, deep NMB effectively reduced postoperative pain after RATT in the current study.

The benefits of deep NMB have been demonstrated in a variety of surgeries. According to a meta-analysis [11] on the effects of deep NMB in laparoscopic abdominal surgery, it was proven that deep NMB reduces early postoperative pain and improves the surgical condition during laparoscopic abdominal surgery. Additionally, two randomized, controlled trials [25,26] reported that deep NMB improves surgical condition during robot-assisted radical prostatectomy. However, to our knowledge, there has been no study that investigated the effect of deep NMB in RATT. This study revealed the effect of deep NMB in reducing postoperative pain after RATT and showed that deep NMB could be beneficial in RATT. Considering that postoperative pain delays recovery and discharge [27], maintaining deep NMB during RATT may improve postoperative recovery, which should be demonstrated in further studies.

We could not demonstrate that deep NMB reduces paresthesia or hypoesthesia after RATT. Considering that the deep NMB group showed a trend of less paresthesia and sensory change, significant results may be obtained if a larger number of participants were investigated, or a longer follow-up was conducted.

This study also demonstrated that deep NMB could be safely maintained during RATT without severe complications or intensive care unit admission. Nevertheless, two cases of postoperative bleeding occurred in the deep NMB group, including the dropped patient. Although we did not observe significant intergroup differences in the incidence of postoperative bleeding, it is noteworthy that the bleeding events occurred only in the deep NMB group. Some studies have reported that sugammadex increases bleeding tendency by prolonging the activated partial thromboplastin time and prothrombin time [28,29,30]. Given that sugammadex was administered at larger doses in the deep NMB group, it is possible that sugammadex played a role in the occurrence of postoperative bleeding.

This study had several limitations. First, most outcomes in this study were subjective indicators. Pain was measured by using the NRS, and paresthesia was assessed by using a questionnaire. The pinprick test relied on the subjective expression of the degree of remnant sensation by patients. Future studies should aim to confirm the effects of deep NMB through more objective indicators. Second, POD 90 data had missing values in 18 patients; hence, more participants are necessary to investigate the effects of deep NMB on chronic pain and sensory changes three months after the surgery. Larger- and longer-scale studies would provide a more accurate evaluation of the effects of deep NMB on chronic symptoms after RATT. Third, we did not measure retractor pressure during surgery. Considering that deep NMB reduced postoperative pain in this study, it could be expected that the retractor pressure would decrease, but we could not prove it. Fourth, we evaluated pain scores as a single assessment. Indicators calculated from repeated pain measurements, such as the sum of pain intensity difference or maximum total pain relief, can be more accurate than single assessments. However, we measured pain scores a total of four times (PACU, POD 1, POD 3, POD 90) during the study period and analyzed their changes by using a linear mixed model.

## 5. Conclusions

Deep NMB showed a significant effect in reducing NRS pain scores after RATT compared to moderate NMB. However, this study could not demonstrate that deep NMB reduces paresthesia or sensory changes after the surgery. Deep NMB may be beneficial in reducing postoperative pain in RATT.

## Figures and Tables

**Table 1:** Baseline demographic and clinical characteristics.

	Moderate NMB (n = 44)	Deep NMB (n = 43)	*p*-Value
Sex (female)	41 (93.2)	40 (93.0)	>0.999
Age (years)	36.3 ± 9.0	35.8 ± 9.1	0.795
BMI (kg/m^2^)	22.9 ± 2.9	22.6 ± 3.4	0.663
ASA class			0.151
1	15 (34.1)	22 (51.2)	
2	25 (56.8)	20 (46.5)	
3	4 (9.1)	1 (2.3)	
Hypertension	0 (0)	1 (2.3)	0.494
DM	0 (0)	1 (2.3)	0.494
Old TB	0 (0)	1 (2.3)	0.494
HBV	1 (2.3)	1 (2.3)	>0.999
Dyslipidemia	1 (2.3)	1 (2.3)	>0.999
Asthma	3 (6.8)	1 (2.3)	0.616
HCD	3 (6.8)	2 (4.7)	>0.999

Values are mean ± standard deviation or number of patients (%). NMB: neuromuscular block; ASA: American Society of Anesthesiologists; BMI: body mass index; DM: diabetes mellitus; TB: tuberculosis; HBV: hepatitis B virus; HCD: herniated cervical disc.

**Table 2:** Operation type, surgeon, tumor size, pathologic result, and TNM stage.

	Moderate NMB (n = 44)	Deep NMB (n = 43)	*p*-Value
Operation type			0.730
HT	2 (4.5)	4 (9.3)	
HT with CCND	36 (81.8)	33 (76.7)	
BTT with CCND	6 (13.6)	6 (14.0)	
Surgeon			0.800
Dr. Nam	26 (59.1)	28 (65.1)	
Dr. Kang	16 (36.4)	14 (32.6)	
Dr. Lee	2 (4.6)	1 (2.3)	
Tumor size (cm)	0.9 ± 0.8	1.4 ± 1.5	0.052
Pathology			0.069
Benign	4 (9.1)	5 (11.6)	
PTC, conventional	40 (90.9)	32 (74.4)	
PTC, follicular variant	0 (0)	4 (9.3)	
PTC, hobnail variant	0 (0)	1 (2.3)	
FTC	0 (0)	1 (2.3)	
TNM stage ^1^			
T stage			0.037
T1	39 (97.5)	33 (86.8)	
T2	0 (0)	4 (10.5)	
T3a	0 (0)	1 (2.6)	
T3b	1 (2.5)	0 (0)	
N stage			0.901
N0	28 (70.0)	25 (65.8)	
N1a	11 (27.5)	12 (31.6)	
N1b	1 (2.5)	1 (2.6)	
M stage			>0.999
M0	40 (100)	38 (100)	

^1^ Data for the TNM stage consisted of 78 patients (Moderate NMB = 40, Deep NMB = 38). Values are mean ± standard deviation, or number of patients (%). NMB: neuromuscular block; HT: hemithyroidectomy; BTT: bilateral total thyroidectomy; CCND: central compartment neck dissection; PTC: papillary thyroid carcinoma; FTC: follicular thyroid carcinoma; TNM: tumor–node–metastasis.

**Table 3:** Perioperative data.

	Moderate NMB (n = 44)	Deep NMB (n = 43)	*p*-Value
Operation time (min)	99 ± 16	102 ± 24	0.577
Anesthesia time (min)	130 ± 17	132 ± 23	0.651
Fluid input (mL)	550 ± 117	564 ± 175	0.664
Propofol (mg)	101 ± 16	104 ± 15	0.398
Rocuronium (mg)	57 ± 9	110 ± 28	<0.001
Remifentanil (μg)	414 ± 132	416 ± 127	0.959
Sugammadex (mg)	121 ± 17	244 ± 39	<0.001
Blood Pressure (mmHg)			
Baseline	89 ± 13	90 ± 12	0.731
Before incision	72 ± 11	75 ± 10	0.331
Docking robot	75 ± 9	78 ± 9	0.201
Undocking robot	69 ± 9	70 ± 7	0.364
Heart Rate (beats/min)			
Baseline	78 ± 13	74 ± 13	0.167
Before incision	86 ± 12	85 ± 12	0.661
Docking robot	79 ± 12	81 ± 10	0.342
Undocking robot	72 ± 11	77 ± 9	0.021
Peak airway pressure (cm H_2_O)			
Before incision	13 ± 2	14 ± 2	0.211
Docking robot	14 ± 1	15 ± 2	0.416
Undocking robot	13 ± 1	13 ± 2	0.655
TOF 0.9 time (s)	99 ± 43	147 ± 78	0.001
PACU time (min)	39 ± 15	39 ± 12	0.852
Hospital stay (days)	4.1 ± 0.5	4.0 ± 0.4	0.353
ICU admission	0 (0)	0 (0)	>0.999
Respiratory failure	0 (0)	0 (0)	>0.999
Desaturation	0 (0)	0 (0)	>0.999
Postoperative bleeding	0 (0)	1 (2.3)	0.494
Rescue analgesic			
PACU	21 (47.7)	19 (44.2)	0.740
POD 1	9 (20.5)	7 (16.3)	0.615
POD 3	1 (2.3)	0 (0)	>0.999
PONV (0–3)			
PACU	0.6 ± 0.9	0.4 ± 0.8	0.398
POD 1	0.0 ± 0.3	0.1 ± 0.4	0.510
POD 3	0.0 ± 0.2	0.0 ± 0.0	0.323

Values are mean ± standard deviation, or number of patients (%). NMB: neuromuscular block; TOF: train-of-four; PACU: post-anesthesia care unit; ICU: intensive care unit; PONV: postoperative nausea and vomiting; POD: postoperative day.

## Data Availability

The data presented in this study are available on request from the corresponding author.

## Supplementary Materials

The following supporting information can be downloaded at: https://www.mdpi.com/article/10.3390/jcm12113633/s1, Table S1: Postoperative pain scores; Table S2: Numbers of patients with severe pain.
