# Supplementary material for: Effects of Deep Neuromuscular Block during Robot-Assisted Transaxillary Thyroidectomy: A Randomized Controlled Trial
Source: J Clin Med. 2023 May 23;12(11):3633. doi: 10.3390/jcm12113633 (PMC10253374; doi:10.3390/jcm12113633)
Supplement: Supplementary file 1 [file jcm-12-03633-s001.zip › jcm-2275179-supplementary.pdf]

Supplementary Table S1. Postoperative pain scores

	Moderate NMB (n = 44)	Deep NMB (n = 43)	Mean difference (95% CI)	Effect size	P-value
Chest pain (0-10)					
PACU	4.3 ± 2.6	3.0 ± 2.4	-1.3 (-2.3, -0.2)	0.510	0.020
POD 1	3.6 ± 2.0	1.6 ± 2.0	-2.0 (-2.9, -1.2)	1.004	<0.001
POD 3	2.0 ± 2.1	1.0 ± 1.3	-1.0 (-1.8, -0.3)	0.596	0.007
POD 90	1.8 ± 2.4	1.4 ± 2.3	-0.4 (-1.5, 0.7)	0.163	0.500
Neck pain (0-10)					
PACU	4.6 ± 2.3	4.0 ± 2.1	-0.6 (-1.5, 0.4)	0.266	0.218
POD 1	3.3 ± 2.2	1.4 ± 1.7	-1.9 (-2.7, -1.0)	0.933	<0.001
POD 3	1.7 ± 2.0	1.1 ± 1.1	-0.6 (-1.3, 0.1)	0.366	0.091
POD 90	1.3 ± 2.1	1.1 ± 1.7	-0.2 (-1.1, 0.8)	0.083	0.733
Axilla pain (0-10)					
PACU	5.9 ± 2.3	5.6 ± 2.1	-0.4 (-1.3, 0.6)	0.170	0.429
POD 1	4.0 ± 2.2	2.4 ± 1.7	-1.5 (-2.4, -0.7)	0.765	0.001
POD 3	2.3 ± 1.8	1.7 ± 1.7	-0.6 (-1.3, 0.2)	0.314	0.148
POD 90	1.1 ± 1.8	1.5 ± 2.0	0.4 (-0.5, 1.3)	0.210	0.387

Values are expressed as mean ± standard deviation. Pain scores were expressed on the numeric rating scale of 0–10 (0 = no pain and 10 = severe pain). NMB, neuromuscular block; PACU, post-anesthesia care unit; POD, postoperative day.

Supplementary Table S2. Numbers of patients with severe pain

	Moderate NMB (n = 44)	Deep NMB (n = 43)	P-value
Severe chest pain			
PACU	13 (29.5%)	5 (11.6%)	0.039
POD 1	4 (9.1%)	1 (2.3%)	0.360
POD 3	1 (2.3%)	0 (0%)	>0.999
POD 90	2 (5.6%)	2 (6.1%)	>0.999
Severe neck pain			
PACU	10 (22.7%)	6 (14.0%)	0.291
POD 1	3 (6.8%)	1 (2.3%)	0.616
POD 3	1 (2.3%)	0 (0%)	>0.999
POD 90	2 (5.6%)	0 (0%)	0.494
Severe axilla pain			
PACU	19 (43.2%)	13 (30.2%)	0.210
POD 1	6 (13.6%)	0 (0%)	0.026
POD 3	1 (2.3%)	0 (0%)	>0.999
POD 90	0 (0%)	0 (0%)	-

Values are expressed as number of patients (%). Pain with a numeric rating scale above 6 was classified as severe pain. The data for POD 90 consisted of 69 patients (Moderate NMB = 36, Deep NMB = 33). NMB, neuromuscular block; PACU, post-anesthesia care unit; POD, postoperative day.
